# Clinical Significance of Biochemical Pregnancy Loss in Recurrent Pregnancy Loss Patients: Insights From Euploid Embryo Transfers Minimizing Embryonic Bias

**DOI:** 10.1002/rmb2.12668

**Published:** 2025-07-23

**Authors:** Yoshimitsu Kuwabara, Tatsunori Shiraishi, Ryoko Kato, Shigeru Matsuda, Akiko Sakata, Yumene Kubota, Ryoko Yokote, Kimihiko Nakao, Mirei Yonezawa, Tomoko Ichikawa, Toshiyuki Takeshita, Shunji Suzuki

**Affiliations:** ^1^ Department of Obstetrics and Gynecology Nippon Medical School Tokyo Japan

**Keywords:** biochemical pregnancy loss, endometrial receptivity, euploid embryo transfer, preimplantation genetic testing for aneuploidy, recurrent pregnancy loss

## Abstract

**Purpose:**

To evaluate the clinical relevance of biochemical pregnancy loss (BPL) in recurrent pregnancy loss (RPL) patients, using data from preimplantation genetic testing for aneuploidy (PGT‐A) to minimize embryonic factors.

**Methods:**

This retrospective cohort study included 52 PGT‐A cycles (48 patients) with single euploid embryo transfers between April 2020 and December 2022. Patients were stratified into three groups: Group A (ART failure without RPL, 18 cycles/17 patients), Group B (RPL following ART pregnancies, 12 cycles/10 patients), and Group C (RPL following natural pregnancies, 22 cycles/21 patients). This classification aimed to assess maternal factors contributing to BPL across different clinical backgrounds. The incidence of BPL, clinical pregnancy rate, and predictive performance of ART outcomes were analyzed, with and without BPL included, using ROC curve analysis.

**Results:**

Biochemical pregnancy loss occurred in 0% (A), 25.0% (B), and 37.5% (C) of patients (*p* = 0.037). Incorporating BPL into miscarriage history significantly improved ART outcome prediction (AUC 0.871 vs. 0.759).

**Conclusion:**

Biochemical pregnancy loss after euploid embryo transfer likely reflects maternal or endometrial pathology. Incorporating BPL into the diagnostic criteria for RPL may enhance clinical assessment and personalized care.

## 1 Introduction

Biochemical pregnancy loss (BPL) refers to a very early pregnancy loss characterized by the detection of β‐human chorionic gonadotropin (β‐hCG) in serum or urine without subsequent confirmation of a gestational sac on ultrasound. In such cases, menstruation‐like bleeding typically follows, marking the spontaneous resolution of the pregnancy at a preclinical stage. Although distinct from clinical miscarriage, BPL constitutes a form of early pregnancy loss that has attracted increasing attention in reproductive medicine.

Definitions and diagnostic criteria for BPL remain inconsistent across clinical guidelines and research frameworks. The European Society of Human Reproduction and Embryology (ESHRE) has advocated for the inclusion of BPL in the diagnostic criteria for recurrent pregnancy loss (RPL), underscoring its potential prognostic significance [1]. In contrast, the American Society for Reproductive Medicine (ASRM) adopts a more conservative approach, restricting the definition of RPL to losses of pregnancies verified by ultrasound or histopathological examination [2]. In Japan, prevailing clinical practice generally follows the ASRM definition. The Japan Society of Obstetrics and Gynecology (JSOG), for example, does not currently recognize BPL as a diagnostic element for RPL, as indicated in its terminology guidelines.

The absence of a universally accepted definition for early pregnancy loss poses challenges for both clinical practice and research. Some experts, such as Simpson (1990) and de Ziegler and Frydman (2021) [3, 4], have included biochemical pregnancies in their definitions of early miscarriage, reporting loss rates of approximately 12%–15%, predominantly before 8–9 weeks of gestation. Conversely, others such as Freedman and Schlaff (2021) [5] have excluded BPL from pregnancy loss statistics by focusing solely on clinically established pregnancies. This lack of consensus limits cross‐study comparability and obscures the true incidence and implications of early pregnancy loss.

In the field of assisted reproductive technology (ART), however, BPL has become an increasingly recognized phenomenon. Advances in sensitive biochemical assays and the widespread use of ART have enabled the routine detection and reporting of biochemical pregnancies during treatment cycles. While ART‐specific datasets frequently include BPL in clinical outcome measures, its true clinical significance—particularly following euploid embryo transfer—remains an area of ongoing debate. Furthermore, inconsistent reporting practices in general clinical and epidemiological contexts contribute to the underestimation of BPL incidence and hinder the development of standardized diagnostic and management strategies.

There is growing recognition that BPL is a biologically and clinically meaningful outcome, rather than a trivial or incidental laboratory finding. Recent evidence suggests that BPL involves multiple interrelated pathways [6]. Several studies have reported that endometrial dysfunction may predispose individuals to pregnancy loss through impaired decidualization [7]. Suboptimal endometrial thickness, impaired blood flow, and residual inflammation following endometritis treatment have been reported to increase the risk of BPL, suggesting that endometrial factors are particularly influential [8]. These observations collectively indicate that BPL may reflect subtle disturbances in maternal reproductive competence and justify its closer clinical scrutiny.

This study examined the clinical implications of BPL in RPL patients who underwent preimplantation genetic testing for aneuploidy (PGT‐A). By eliminating embryonic aneuploidy as a confounding factor using single euploid embryo transfers, we aimed to clarify whether BPL should be included in the miscarriage count and considered a clinically significant event in reproductive care. We hypothesized that maternal background factors may differ between these groups. By stratifying patients according to reproductive history—infertility without RPL (Group A), RPL after ART pregnancies (Group B), and RPL after natural pregnancies (Group C)—we aimed to explore whether distinct maternal factors contribute to BPL occurrence even after euploid embryo transfer.

## 2 Materials and Methods

### 2.1 Study Population

This retrospective study was conducted at a single fertility center and included patients who underwent preimplantation genetic testing for aneuploidy (PGT‐A) followed by single euploid blastocyst transfer between April 2020 and December 2022.

A total of 100 patients underwent PGT‐A during the study period. Of these, 55 patients (59 embryo transfer cycles) proceeded to single euploid embryo transfer. Cases involving poor‐quality embryos—including Day 7 blastocysts, embryos derived from in vitro matured oocytes, and embryos with grade C morphology—were excluded. Ultimately, 48 patients (52 embryo transfer cycles) who received Day 5–6 blastocysts graded 3BB or higher were included in the final analysis.

### 2.2 Ovarian Stimulation and Oocyte Retrieval

Controlled ovarian stimulation was performed using a GnRH antagonist protocol. Recombinant follicle‐stimulating hormone (rFSH; Gonal‐F) was administered at a dose of 150–225 IU/day starting from Day 2 or 3 of the menstrual cycle. Final oocyte maturation was triggered with either 10,000 IU of human chorionic gonadotropin (hCG) or 0.2 mg of triptorelin when at least two follicles reached ≥ 18 mm in diameter. Oocyte retrieval was performed 36 h after the trigger, under transvaginal ultrasound guidance.

### 2.3 Embryo Culture and PGT‐A

The retrieved oocytes were fertilized via intracytoplasmic sperm injection (ICSI) and cultured to the blastocyst stage. Trophectoderm biopsy was performed on Day 5 or 6 on blastocysts with a morphology grade ≥ 3BB, according to the Gardner grading system. Embryos with poor morphology (grade C), Day 7 development, or of in vitro matured (IVM) origin were excluded. PGT‐A was performed using next‐generation sequencing (NGS). All biopsies were conducted by a single experienced embryologist.

### 2.4 Endometrial Preparation and Embryo Transfer

Frozen–thawed embryo transfers were carried out in the hormone replacement therapy (HRT) cycles. Endometrial preparation was initiated on cycle Day 2 or 3 using transdermal estradiol patches (Estrana tape, typically 2.16–3.6 mg/day), with or without the addition of oral estradiol valerate (Jurin) as needed. When endometrial thickness reached ≥ 8 mm and serum estradiol levels (E2) exceeded 180 pg/mL, vaginal micronized progesterone (Utrogestan, 600 mg/day) was initiated. A single euploid embryo was transferred on Day 6 of progesterone exposure. All embryo transfers were performed following a standardized clinical protocol and managed by a single experienced medical team to minimize variability in laboratory and clinical procedures.

### 2.5 Pregnancy Outcome Definitions

Serum β‐hCG levels were measured 7 days after embryo transfer to detect early implantation.

Biochemical pregnancy was defined as a serum β‐hCG level > 5 IU/L without ultrasound evidence of a gestational sac.

Clinical outcomes were categorized into the following groups:

- hCG‐negative: No rise in β‐hCG following embryo transfer.
- Biochemical pregnancy: Defined above.
- Clinical pregnancy: Confirmation of a gestational sac by ultrasound.

Ongoing pregnancy was defined as the presence of a fetal heartbeat beyond 12 weeks of gestation.

### 2.6 RPL Risk Assessment and Treatment

All RPL patients underwent standardized evaluation that included antiphospholipid antibody testing (lupus anticoagulant, anticardiolipin, and anti‐β2GPI), antinuclear antibody (ANA), thyroid function (TSH and free T4), and thrombophilia screening. The thrombophilia workup comprised the antigen and activity levels of protein S and protein C, as well as antithrombin III and platelet aggregation testing. Uterine anomalies were assessed using three‐dimensional transvaginal ultrasonography, which enabled a detailed morphological evaluation. When abnormalities were identified, individualized treatments were implemented in accordance with current clinical guidelines, including low‐dose aspirin, unfractionated heparin, or levothyroxine, as appropriate.

### 2.7 Statistical Analysis

All statistical analyses were performed using SPSS version 26.0 (IBM Corp., Armonk, NY, USA). Continuous variables were expressed as mean ± standard deviation (SD) and compared using the *t* test or the Mann–Whitney *U* test, as appropriate based on the data distribution. Categorical variables were compared using the chi‐squared test or Fisher's exact test. For comparisons involving more than two groups, either one‐way analysis of variance (ANOVA) with Bonferroni correction or the Kruskal–Wallis test followed by post hoc pairwise Mann–Whitney *U* tests (with Bonferroni correction) was applied, depending on the data distribution. Receiver operating characteristic (ROC) curve analysis was conducted using DATAtab to evaluate the predictive performance of previous miscarriage counts, with or without the inclusion of BPL. A *p*‐value of < 0.05 was considered statistically significant.

## 3 Results

### 3.1 Patient Selection and Classification

**FIGURE 1:**
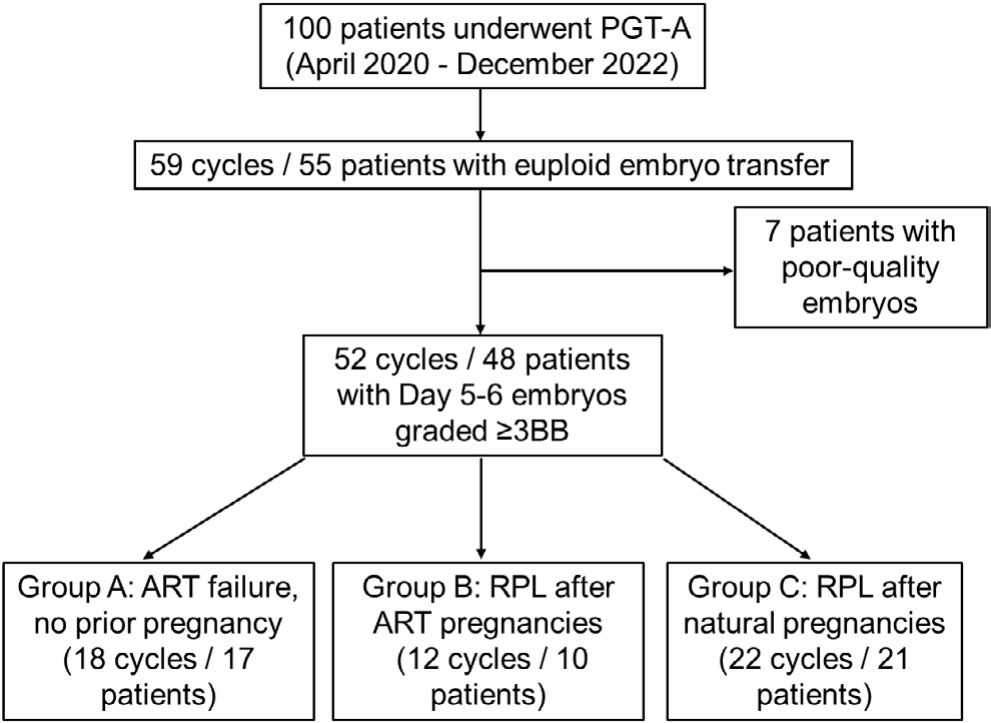
Group‐wise incidence of BPL. Bar graph showing BPL occurrence by patient group: 0% in Group A, 25% in Group B, and 37.5% in Group C. The difference in BPL rates across groups was statistically significant (*p* = 0.037).

As illustrated in Figure 1, the final study cohort comprised 48 patients (52 cycles) who underwent single euploid embryo transfer. These patients were stratified into three distinct groups according to their reproductive histories, allowing for subgroup comparisons to elucidate clinical outcomes and potential contributing factors:

- Group A (ART failure without RPL): Patients undergoing ART for primary or secondary infertility without a history of recurrent pregnancy loss (defined as fewer than two prior spontaneous miscarriages).
- Group B (RPL after ART pregnancies): Patients who experienced recurrent pregnancy loss following ART‐derived pregnancies.
- Group C (RPL after natural pregnancies): Patients with recurrent pregnancy loss following natural conception.

Although Group A may include patients with one previous miscarriage or biochemical pregnancy, none met the diagnostic criteria for RPL. Thus, this group primarily represents infertile patients without a significant history of pregnancy loss. This classification provided a framework to investigate differences in biochemical pregnancy loss and related outcomes based on the patients' prior reproductive experiences.

### 3.2 Baseline Characteristics

Baseline characteristics (Table 1) showed similar distributions of age, body mass index (BMI), and parity across the three groups: Group A (18 cycles/17 patients), Group B (12 cycles/10 patients), and Group C (22 cycles/21 patients). However, the number of previous miscarriages was significantly lower in Group A (ART failure without prior pregnancies) compared to Groups B and C (RPL after ART and natural pregnancies, respectively) (*p* < 0.05), consistent with their clinical backgrounds.

**TABLE 1:** Patient demographics.

Group (cycles/patients)	Age (mean ± SD)	BMI (kg/m^2^)	Previous miscarriages	Parity*	Endometrial thickness (mm)*	Estradiol (pg/mL)
A: ART failure (18/17)	34.1 ± 2.5	22.7 ± 3.0	0.47 ± 0.38	0.3 ± 0.5	10.3 ± 1.37	421.33 ± 163.19
B: RPL after ART (12/10)	35.2 ± 2.7	22.7 ± 3.6	2.6 ± 0.71	0.4 ± 0.6	8.74 ± 1.1	349.53 ± 129.04
C: RPL after natural (22/21)	35.5 ± 2.9	22.3 ± 2.8	3.19 ± 1.26	0.4 ± 0.6	9.73 ± 1.99	403.82 ± 174.93

*Note:* Summary of baseline characteristics including age, parity, number of previous miscarriages, endometrial thickness, and serum estradiol levels across Groups A–C.

* Statistically significant differences among groups were detected (*p* < 0.05).

At the time of embryo transfer, mean endometrial thickness differed significantly among groups: 10.3 ± 1.4 mm in Group A, 8.7 ± 1.1 mm in Group B, and 9.7 ± 2.0 mm in Group C (*p* = 0.020). Serum estradiol levels on the day progesterone was initiated did not show significant differences (*p* = 0.516), suggesting that hormonal priming was comparable. Despite the slight variation in endometrial thickness, values remained within acceptable clinical ranges, indicating that endometrial preparation was largely uniform.

### 3.3 Recurrent Pregnancy Loss Risk Factors

Table 2 summarizes the RPL risk factors in Groups B and C. Over 75% of patients in both groups had at least one identified abnormality, such as antiphospholipid antibodies, uterine anomalies, thyroid dysfunction, or thrombophilic conditions, and no significant differences were noted between the groups. These results highlight the substantial burden of maternal risk factors in RPL patients regardless of the type of previous pregnancies.

**TABLE 2:** RPL‐related risk factors.

Risk factor	Total (*n* = 31), number (%)	Group B (*n* = 10), number (%)	Group C (*n* = 21), number (%)
Antiphospholipid antibody (+)	2 (6.5)	1 (10)	1 (4.8)
Uterine anomaly	1 (3.2)	0 (0)	1 (4.8)
Antinuclear antibody	4 (12.9)	1 (10)	3 (14.3)
Thyroid dysfunction	5 (16.1)	1 (10)	4 (19)
Factor XII deficiency	7 (22.6)	4 (40)	3 (14.3)
Protein C antigen ↓	0 (0)	0 (0)	0 (0)
Protein C activity ↓	0 (0)	0 (0)	0 (0)
Protein S antigen ↓	0 (0)	0 (0)	0 (0)

*Note:* Proportion of patients in Groups B and C testing positive for recurrent pregnancy loss‐associated risk factors. No significant group differences were observed. Over 75% of patients in both groups had at least one positive finding and standard therapy was introduced according to the type of abnormality.

Standardized treatments were applied according to detected abnormalities: levothyroxine for thyroid dysfunction, unfractionated heparin with low‐dose aspirin for antiphospholipid antibody syndrome, and low‐dose aspirin alone for other thrombophilic tendencies and platelet aggregation abnormalities.

### 3.4 Clinical Outcomes and Incidence of BPL

Table 3 presents the clinical outcomes. BPL occurred in 0.0% of Group A, 25.0% of Group B, and 37.5% of Group C (*p* = 0.037), demonstrating a stepwise increase. Notably, BPL was absent in Group A under conditions of euploid embryo transfer and consistent laboratory protocols, suggesting that maternal background, rather than embryo chromosomal status, may play a key role in BPL pathogenesis.

**TABLE 3:** Pregnancy outcomes.

Outcome	Group A (%)	Group B (%)	Group C (%)
Biochemical pregnancy loss (BPL)*	0.0	25.0	37.5
Clinical pregnancy	66.7	50.0	45.5
Ongoing pregnancy	66.7	50.0	40.0

* Statistically significant difference among groups (*p* = 0.037).

The clinical pregnancy rates were 66.7% in Group A, 50.0% in Group B, and 45.5% in Group C. Ongoing pregnancy rates followed a similar pattern, with the highest rate in Group A (66.7%) and lower rates in Groups B (50.0%) and C (40.0%). Chi‐square analysis showed no significant differences among groups in clinical pregnancy rates (*p* = 0.33) or ongoing pregnancy rates (*p* = 0.21). Pairwise comparisons between Groups A and C also showed no significant differences (clinical pregnancy: *p* = 0.22; ongoing pregnancy: *p* = 0.11). Furthermore, a pairwise comparison of BPL rates between Groups B and C showed no significant difference (*p* = 0.528, Fisher's exact test).

These data indicate that even with euploid embryos, patients with a history of RPL—particularly after natural pregnancies—may remain at increased risk for early pregnancy failure, including BPL.

### 3.5 Relationship Between Prior Reproductive History and Pregnancy Outcomes

Table 4 compares the reproductive histories of patients who experienced BPL, clinical pregnancies, and hCG‐negative outcomes. Patients in the BPL group had significantly more previous miscarriages (3.57 ± 1.27 vs. 2.44 ± 0.81, *p* < 0.05) and previous BPL episodes (1.14 ± 0.38 vs. 0.38 ± 0.62, *p* < 0.05) compared with the clinical pregnancy group. In contrast, no significant differences were observed between the clinical pregnancy group and the hCG‐negative group for either prior miscarriage counts or prior BPL episodes. Similarly, no significant differences were observed between the BPL group and the hCG‐negative group for these parameters.

**TABLE 4:** Prior pregnancy history by outcome.

Outcome	Previous miscarriages (mean ± SD)	Prior BPL episodes (mean ± SD)
hCG‐negative	3.00 ± 0.76	0.63 ± 0.74
Biochemical pregnancy	3.57 ± 1.27*	1.14 ± 0.38^†^
Clinical pregnancy	2.44 ± 0.81*	0.38 ± 0.62^†^

*Note:* Comparison of previous miscarriage count and prior BPL episodes between patients who experienced BPL and those who achieved clinical pregnancy or hCG‐negative in the current cycle. BPL group had significantly greater prior reproductive loss burden. This analysis includes only patients in Groups B and C (RPL after ART or natural pregnancies), as Group A (without RPL history) was not applicable for prior pregnancy history evaluation.

* *p* < 0.05 vs. clinical pregnancy.

^†^ *p* < 0.05 vs. clinical pregnancy.

### 3.6 Predictive Value of Incorporating BPL

**FIGURE 2:**
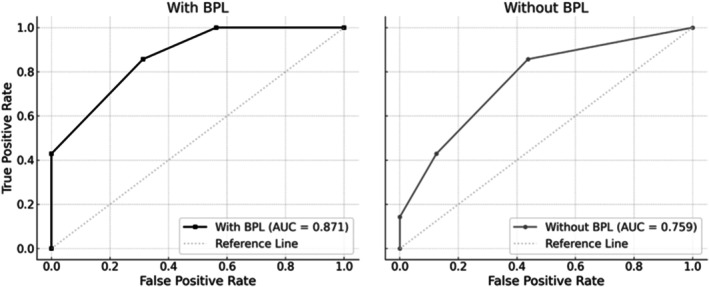
ROC curve for ART outcome prediction. ROC curves comparing prediction models for ART outcome with and without inclusion of BPL in the count of previous pregnancy losses. Including BPL improved the AUC from 0.759 to 0.871, enhancing predictive performance.

Figure 2 presents the ROC curve analysis evaluating the predictive performance of prior pregnancy loss counts in relation to ART outcomes. When BPL was excluded, a cutoff of three previous losses yielded a sensitivity of 85.7%, specificity of 62.5%, PPV of 46.0%, and NPV of 90.0%, with an AUC of 0.759.

In contrast, including BPL in the total count and applying a cutoff of four losses maintained the sensitivity (85.7%) but improved specificity (68.7%), with the PPV rising to 73.2% and NPV to 91.7% (AUC 0.871).

These findings suggest that incorporating BPL into the reproductive history enhances risk stratification. In particular, the improved specificity and positive predictive value indicate its potential role in identifying patients at higher risk for ART failure, reinforcing its clinical and prognostic importance.

## 4 Discussion

Our findings suggest that BPL is not merely a by‐product of embryonic aneuploidy but may also reflect underlying maternal factors, especially in patients with RPL. This interpretation is supported by our finding that BPL was present in the RPL groups but absent in patients with prior ART failure, despite all embryos being chromosomally normal.

In this study, stratifying patients according to reproductive history revealed differences in prior pregnancy burden across groups. Group C demonstrated a higher average number of previous miscarriages compared to Group B, suggesting that patients with RPL after natural pregnancies may have more severe underlying maternal or endometrial pathology. Conversely, Group B may include more cases where prior embryonic chromosomal abnormalities in ART cycles led to miscarriages, even though patients subsequently fulfilled RPL criteria. These distinctions may explain the differential incidence of BPL observed across the groups.

Notably, none of the patients in the ART failure group (Group A), who underwent PGT‐A under the same protocol and embryology team during the study period, experienced biochemical pregnancy. The clinical and ongoing pregnancy rates in this group were both 66.7%. Compared to the outcomes reported in the nationwide Japanese PGT‐A registry study conducted by the Japan Society of Obstetrics and Gynecology (JSOG), where the ongoing pregnancy rate was 53.9% for ART failure cases and 60.7% for RPL cases, our ART failure group demonstrated superior results, whereas our RPL cohort showed comparatively lower outcomes [9]. Therefore, the poor ART outcomes observed in the RPL groups are unlikely to be attributed to technical issues related to embryo biopsy, but rather suggest intrinsic maternal factors specific to the RPL population.

It is also important to note that the RPL group in the JSOG database likely included patients with repeated pregnancy losses due to random embryonic aneuploidy, whereas our RPL patients were managed at a tertiary center specializing in RPL, with rigorous diagnostic screening and individualized treatments based on established etiologies. This distinction underscores the clinical relevance of our findings and supports the hypothesis that maternal factors are critical for explaining BPL when chromosomal abnormalities have already been excluded through PGT‐A.

Previous studies have highlighted the embryonic origins of BPL. For instance, Salumets et al. (2006) [10] reported that maternal age was the only predictor of BPL in ART cycles, independent of embryo morphology. Moreover, a Japanese PGT‐A pilot study demonstrated that BPL rates decreased from 45% to 12.5% after aneuploidy screening [9]. However, in our study, BPL rates remained high in patients with RPL despite euploid transfers.

Conversely, several lines of evidence support a maternal contribution to BPL. Kolte et al. (2014) [11] demonstrated that nonvisualized pregnancy losses, including BPL, have prognostic significance in cases of unexplained RPL, supporting the inclusion of BPL in clinical assessments. Based on these findings, the ESHRE guidelines now recommend BPL incorporation in the diagnostic criteria for RPL. Moreover, Dahan et al. (2020) [12] reported that BPL rates remain relatively stable across different maternal ages, in contrast to the age‐related increase observed in clinical miscarriage rates, further reinforcing the notion that BPL is not solely attributable to embryonic quality but may also reflect underlying maternal factors.

Recent studies have supported the notion that BPL results from a complex interplay between endometrial and biochemical factors. An integrated model describes early pregnancy as progressing through four sequential stages: preimplantation (regulated by prolactin and prokineticin‐1) [7], implantation (involving hCG and specific microRNAs) [13], early postimplantation (involving signaling pathways such as leukemia inhibitory factor, epidermal growth factor, Indian hedgehog, and WNT, which regulate trophoblast‐uterine interactions) [14], and maintenance (sustained by hCG and progesterone) [6]. Endometrial dysfunction, particularly impaired decidualization, may create a less selective uterine environment, allowing the implantation of lower‐quality embryos and increasing the likelihood of BPL. Although various biomarkers, including hCG, progesterone, and microRNAs, have been proposed, no single marker has demonstrated a sufficient individual predictive value.

Our ROC analysis clearly demonstrated an improved prediction of ART outcomes when BPL was included in the count of previous losses, supporting its diagnostic and prognostic relevance. These findings argue for reconsideration of current clinical guidelines and encourage a more nuanced understanding of early implantation failure. Incorporating BPL into the count of previous losses enhanced both the specificity and overall predictive accuracy of ART outcome models, while maintaining high sensitivity, further reinforcing its clinical and diagnostic importance.

This study has several limitations. First, its retrospective design and relatively small sample size from a single center may limit the generalizability of the findings. Second, although major chromosomal aneuploidies were filtered out by PGT‐A, other embryonic factors such as epigenetic abnormalities or mitochondrial dysfunction, which are not routinely assessed in clinical practice, may still have contributed to BPL. Third, in Group C, prior BPL episodes were identified based on detailed reproductive history obtained through interviews. As these patients were actively attempting conception under evaluation for RPL, serial pregnancy testing was generally performed with close clinical monitoring. However, unrecognized BPL episodes that occurred prior to medical consultation may have been missed, potentially leading to underestimation of BPL frequency in this group. Fourth, potential confounders such as endometrial receptivity and immunological markers were not uniformly evaluated in all patients. Finally, it should be emphasized that this study exclusively focused on cycles following PGT‐A with euploid embryo transfer. While our findings support the clinical significance of BPL in the context of PGT‐A cycles where embryonic aneuploidy is excluded, it remains uncertain whether similar implications can be meaningfully applied to BPL observed in non‐PGT‐A ART cycles or natural pregnancies, where embryonic factors may still play a certain role. Further large‐scale cohort studies including both PGT‐A and non‐PGT‐A cycles are necessary to clarify this issue.

Despite these limitations, this study has several notable strengths. All PGT‐A cycles were conducted within the same period using a uniform ovarian stimulation protocol, and embryo biopsies were performed by a single experienced embryologist. This consistency minimizes technical variability and allows for meaningful comparisons based purely on patient background. Differences in BPL incidence between the groups could not be explained by variations in endometrial thickness or serum estradiol levels at the time of transfer, both of which were comparable across the groups. This finding supports the hypothesis that BPL reflects deeper endometrial or immunological dysfunction that is not captured by standard morphometric or hormonal parameters. All patients with RPL were evaluated and managed using standardized screening and treatment protocols at a tertiary referral center. This clinical precision enhanced the internal validity and interpretability of the findings, even in the context of a modest cohort size.

Taken together, our findings suggest that BPL, particularly after euploid embryo transfer, is not merely incidental but reflects underlying maternal and endometrial factors relevant to implantation and early pregnancy maintenance. Incorporating BPL into the reproductive history significantly improved the predictive performance for ART outcomes, highlighting its potential diagnostic and prognostic utility. While further validation in larger cohorts is warranted, these findings support the clinical relevance of BPL in guiding prognosis and individualized patient counseling in the management of patients with RPL.

## 5 Conclusion

Biochemical pregnancy loss particularly after euploid embryo transfer, likely reflects maternal factors rather than embryonic abnormalities. Incorporating BPL into the diagnostic framework for RPL enhances clinical assessment, especially when embryonic aneuploidy has been excluded. Furthermore, including BPL in reproductive history improves the predictive performance of ART outcome models. While further validation is warranted, these findings underscore the clinical importance of BPL as both a diagnostic and prognostic marker and for guiding individualized management in RPL.

## Ethics Statement

This study was approved by the Institutional Review Board of Nippon Medical School (Approval No. A‐2019‐011) and was conducted in accordance with the principles of the Declaration of Helsinki. Informed consent was obtained from all participants.

## Conflicts of Interest

The authors declare no conflicts of interest.

## Data Availability

The data that support the findings of this study are available on request from the corresponding author. The data are not publicly available due to privacy or ethical restrictions.
